# Albuminuric Retinitis, and Its Bearing on Prognosis

**Published:** 1908-11-07

**Authors:** 


### SPECIAL ARTICLE.

### ALBUMINURIC RETINITIS, AND ITS BEARING ON PROGNOSIS.

The name " albuminuric " retinitis would naturally suggest that the condition might be expected under any circumstances when there was albuminuria. This, of course, is very far from being the case. There are many albuminuric states in which retinitis is not the least likely to occur. In the albuminuria of adolescence, for example, sometimes called physiological albuminuria; or in cases of albuminuria secondary to failure of the right side of the heart, from chronic valvular disease, cardiac muscular degeneration, chronic bronchitis, emphysema, and so on; or in cases of severe primary anæmia, or in cases of low blood-pressure the result of a preceding fever, or due to some cachexia, and so on. It appears almost, but not quite, essential that there should not only be albuminuria, but also organic disease of the kidney, if albuminuric retinitis is to occur. Nor is it every organic disease of the kidney that may cause such retinitis. It seldom or never occurs, for example, as a result of renal calculus, tuberculous disease of the kidney, or malignant new growths of the organ. Lardaceous disease is not liable to be associated with it, although there is, perhaps, no other affection in which the amount of albumen in the urine may be so large as it sometimes is in lardaceous disease. In those cases of lardaceous disease in which albuminuric retinitis has occurred, it is very probable that there was more than the amyloid disease present; for lardaceous disease of the kidney is apt to be accompanied by actual nephritis.

It is particularly in those forms of albuminuria which are generally called Bright's disease that albuminuric retinitis is to be expected; and of the three main clinical groups of Bright's disease—namely, acute nephritis, chronic tubal nephritis, and granular kidney—it is the last two which are far more commonly associated with albuminuric retinitis than is the first. It must be borne in mind, however, that many a case in which acute nephritis is diagnosed is not really a case of primary acute nephritis; more often than not, especially in adults, there has been an insidious and unsuspected degeneration of the kidneys, and then a sudden acute exacerbation occurs, and attracts attention as though it were the primary attack.

It is not, however, quite true to say that albuminuric retinitis can only occur in Bright's disease. There are a few other conditions in which it may be found. For example, it may arise secondary to a stricture of the urethra, or to an enlargement of the prostate, or to a prolapse of the uterus; for in each of these conditions there will, or may, be chronic obstruction to the outflow of urine, with consequent ascending nephritis. When ascending nephritis is mentioned, the mental picture most apt to be called up is one of "surgical" kidney, or suppurative pyelo-nephritis, and the other form of ascending nephritis is apt to be forgotten. This other form is *chronic* ascending nephritis, without any pyuria, but with gross fibrotic changes in the kidney not very unlike those of granular kidney. The effects upon the heart, upon the blood-vessels, upon the blood-pressure, and so on, are similar to the effects of granular kidney; the condition cannot be included, properly speaking, under the head of Bright's disease, and yet it is a possible cause of albuminuric retinitis.

Again, in some cases of infarction of the kidney, particularly if there be fungating endocarditis, a nephritis may occur around each infarcted area, and albuminuric retinitis is sometimes found.

In some of the primary anæmias, again, especially in pernicious anæmia, not only may hæmorrhages occur, but also actual retinitis similar to that seen in Bright's disease; and yet there need be no real kidney disease in these cases.

Notwithstanding the possibility of getting albuminuric retinitis in a few other conditions besides Bright's disease, as described above, it is pre-eminently in the chronic forms of the latter that it occurs. It is an everyday experience to meet with cases of cyanosis, dropsy, breathlessness, bronchitis, orthopnœa, albuminuria, and so on, in which there is obvious failure of cardiac compensation, but in which it may be very difficult to determine whether the heart failure is due to primary cardiac mischief, to primary lung trouble such as emphysema, or to primary kidney disease such as granular kidney with high blood-pressure. It is true that in all such cases the most important step is to determine whether or not there are a pathological number of renal tube casts in the urine; but not infrequently the number of casts found may still leave the question in doubt, and in any case the examination for casts requires time, a centrifugal machine, and a microscope.

Nor is it only in cases such as the above that the presence of albuminuric retinitis is of diagnostic value. With granular kidney the urine changes are frequently indefinite, even on the most careful investigation. In an elderly gentleman, complaining perhaps of nothing worse than a feeling of not being quite up to the mark, albuminuric retinitis may be almost the only evidence of his condition.

It is not, of course, in every case one comes across that the ophthalmoscope affords valuable information in this way. Nevertheless it is of great value in quite enough cases to make it worth while to make a large number of negative examinations on the chance of finding a positive one, and we have indicated above the kind of cases in which the examination may be invaluable in diagnosis.

The statistics upon the proportion of Bright's disease cases in which albuminuric retinitis occurs are very various, according to the source from which they come. In some hospitals the care with which the fundus oculi is examined is far greater than it is in others, and the proportion of cases in which the retinitis is found is proportionately larger. Moreover, it is not altogether easy to be sure whether a given patient is suffering from chronic tubal nephritis pure and simple, or from a chronic interstitial nephritis (granular kidney) with superposed tubal mischief; whether another patient has acute tubal nephritis pure and simple, or has acute nephritis superposed upon chronic tubal trouble which has hitherto been latent; and so on. It seems clear, however, that the number of cases is small in which albuminuric retinitis develops during acute nephritis such as the post-scarlatinal nephritis of children. In the acute nephritis of pregnancy the retinitis is not so uncommon, but here, almost more so than in any other cases, it is difficult to determine with certainty that there is no chronic nephritis underlying the acute disease set up by pregnancy. At all events, it is particularly in the chronic nephritis cases that albuminuric retinitis is liable to occur; and it is more liable to affect patients with chronic tubal nephritis than it is those with small red granular kidney. The highest figures show that 20 out of 100 patients with chronic Bright's disease have albuminuric retinitis; the lowest figure is 9 per cent. Sutton has analysed a large number of cases, and states that out of every 100 patients with chronic tubal nephritis 14 will have the retinitis; and out of 100 patients with small red granular kidneys about 9 show the retinal changes. The trouble may appear in one eye sooner than in the other, but practically always the mischief is bilateral.

The ophthalmoscopic changes may be restricted to the retina, which is most commonly the case; or may affect both the retina and the optic disc, which is not uncommon; or may be restricted to the optic disc, which is rarest of all. To take the changes that may be seen in the optic disc first, they are similar to those of optic neuritis due to other causes, except that there is less filling up of the physiological cup (" choked disc ") than there is in optic neuritis due to an intracranial tumour. The disc becomes hyperæmic; small vessels at its margin become injected and visible where such vessels are usually not seen; the veins become engorged; the arteries look proportionately smaller; the edges of the disc become blurred, with striations compared to those of a rising sun radiating out from the edge of the disc into the retina; in very severe cases the hyperæmia and the blurring may make it very difficult to distinguish the disc at all from the rest of the fundus. These changes depend upon an interstitial neuritis, with exudation of serum, lymph, and leucocytes, which may be sufficient to cover over the vessels in places and cause apparent interruption of their continuity.

It takes an expert to distinguish albuminuric optic neuritis from optic neuritis from other causes, when there is no accompanying retinitis; it is fortunate, therefore, that the majority of cases show retinal changes as well. These may be of three chief kinds, namely: (1) hæmorrhages, old or recent; (2) atrophy of the retina in patches; and (3) acute inflammation with exudation of lymph as described above for the optic disc. Each of these three gives distinctive appearances. Let us take each in turn.

The hæmorrhages, if recent, are readily detected by their dark red colour and by their proximity to the blood-vessels. It must be remembered that they occur *in the retina*, and not in the optic disc. Hæmorrhages in the optic disc are quite rare, but they may occur in any part of the retina: either near the optic disc or at any distance away from it. These hæmorrhages are often described as " flame-shaped," but there is really nothing particularly characteristic about their outline. They may be any shape and any size, though usually they are not big; they are naturally larger at the spot where the bleeding occurred, and taper off along the vessel from this spot. When the hæmorrhage is old it loses its red colour, becoming a white spot, distinguished from other white spots there may be in the retina by the fact that each is in direct relation to a vessel, whilst the vessel at the same time runs past or through the spot without interruption in continuity.

The atrophy of the retina in patches gives rise to other white spots, which present a bright glistening appearance compared sometimes to the sheen of white satin. These may be dotted or " stippled " all round the optic disc, or they may be restricted to some small area of the retina, such as the fovea centralis. The whiteness of the patches is perhaps due to atrophy not only of the retina but of the choroid coat as well, so that the white sclerotic at the back of the eye is seen through what ought to be retina. These satiny patches are perhaps the most distinctive of all the changes in albuminuric retinitis; they differ from the atrophic patches seen in syphilitic choroiditis in that there is little or no black pigment around them, and they differ from old hæmorrhages in having no particular relationship to the blood-vessels.

The acute inflammatory changes in the retina are less often seen than are either the hæmorrhages or the satiny white patches; the exudation of lymph and leucocytes, occurring as it does from within the vessels, first gives rise to a white border along each side of the latter, and presently the lymph accumulates more in one place than another, burying the vessels, and causing an appearance of white patches into which vessels run at one side, get lost to sight, and emerge again on the other side. The optic disc is always involved in such cases, and may be almost indistinguishable from the rest of the retina. The acute inflammatory retinitis is, however, less often seen than are the chronic changes which are equally diagnostic—namely, the old and recent hæmorrhages and the white patches of choroido-retinal atrophy.

It will be noted that white patches of at least three kinds may be observed in albuminuric retinitis, namely: (1) Old hæmorrhages; (2) sheeny patches of retinal atrophy; (3) patches of exuded lymph.

In a certain proportion of cases the retinal changes may cause impairment of vision; in a few cases this impairment is very great; in a great many cases, however, there is no impairment of vision at all. One must not wait until the patient complains about his sight before one makes the ophthalmoscopic examination. The amount of impairment of vision depends at least partly upon whether or not the hæmorrhages, atrophic spots, and exudation involve the macula lutea; it is quite possible, and even relatively common, to have much change in the retina, and yet so little actually at the macula that vision may remain good.

The prognosis as regards vision has been touched upon above; that is to say, vision need not necessarily be impaired at all. If there should be impairment of vision, it may be much or little. In a few cases, having been bad, vision has improved again, and even recovered completely; much more frequently, however, sight gets worse instead of better, and yet it is distinctly rare for the patient to become stone blind. The patient will in all probability always be able to distinguish light from dark, even if no more be possible; in these unhappy cases it is a certain amount of consolation to be assured of this.

The prognosis as regards life is bad. It is true that cases are on record where albuminuric retinitis has been known to be present for as long as 15 years before death; and it is also true that some cases of similar retinitis, in association with the nephritis of pregnancy or with undoubted acute without chronic fatal nephritis, have been recorded as recovering completely as the albuminuria disappeared; but upon the whole, and as much in relation to pregnancy as in connection with other cases of nephritis, the prognosis is bad. Nevertheless, from collections of several hundreds of cases, it is clear that the chances are that a patient with albuminuric retinitis will not live one year; many die within a few months of the discovery of the retinitis; far less than half live on to the end of the second year; and only a few rare cases live longer than that.

